# Mud Therapy Combined with Core Exercise for Chronic Nonspecific Low Back Pain: A Pilot, Single-Blind, Randomized Controlled Trial

**DOI:** 10.1155/2020/7547452

**Published:** 2020-04-03

**Authors:** Suk-Chan Hahm, Ho-Jin Shin, Min-Goo Lee, Sung Jae Lee, Hwi-Young Cho

**Affiliations:** ^1^Graduate School of Integrative Medicine, CHA University, Seongnam, Republic of Korea; ^2^Department of Physical Therapy, Gachon University, Incheon, Republic of Korea; ^3^Department of Physiology, Korea University College of Medicine, Seoul, Republic of Korea; ^4^Department of Integrative Medicine, Korea University College of Medicine, Seoul, Republic of Korea

## Abstract

**Background:**

Low back pain (LBP) is common in the elderly and an appropriate intervention for LBP management should be investigated. The aim of this study is to investigate the potential of mud-heat intervention combined with core exercise as an alternative intervention for relieving pain and improving motor function in individuals with nonspecific chronic LBP.

**Methods:**

Thirty-one individuals with chronic nonspecific LBP were randomly allocated to either the intervention group (*n* = 16) or the control group (*n* = 15). The intervention group used a mud pack for 30 min and performed a core-exercise program for 50 min twice a day for 4 days (8 sessions). The control group performed the core-exercise program only, at the same time point as the intervention group. Pain intensity was assessed using a 100 mm visual analog scale and a pain pressure threshold (PPT) as the primary outcomes. The secondary outcome measures included functional disability by LBP (Oswestry Disability Index), muscle properties, and static/dynamic balance.

**Results:**

There was a significant group difference in pain intensity at rest (*p*=0.048) and in the PPT at the two sites assessed (2 cm lateral to L3 spinous process, *p*=0.045; 2 cm lateral to L5 spinous process, *p*=0.015). No group differences were found in terms of muscle properties. Compared to core exercise only, moor-heat therapy and core exercise showed a significant improvement in static balance (*p*=0.026) and dynamic balance (*p*=0.019).

**Conclusion:**

Mud therapy combined with core exercise is effective in relieving pain and improving motor function in patients with chronic nonspecific LBP. Further research is needed to underpin these preliminary results.

## 1. Background

Low back pain (LBP) is very common, with an estimated 40% to 80% of individuals experiencing LBP over the course of their lives [1]. The majority of LBP is nonspecific (approximately 90% of cases), and nonspecific LBP is a common health problem that most people of all ages suffer from at some point [2]. It is known that nonspecific LBP is associated with a decreased range of motion (ROM), muscle strength, and flexibility of the lumbar and hip joints [3], which can result in tension, soreness, and stiffness. These changes induced by nonspecific LBP can lead to functional limitations in daily living activities and a decrease in quality of life [4].

Currently, there are no interventions to cure chronic LBP, but there are both pharmacologic and nonpharmacologic interventions for the improvement of LBP and its related functional problems [2]. The commonly prescribed medications for LBP management are nonsteroidal anti-inflammatory drugs (NSAIDs), skeletal muscle relaxants, and opioid analgesics [5, 6]; however, adverse effects related to the long-term use of these medications have been reported [2]. A previous Cochrane review reported that opioids only have short-term effectiveness on chronic LBP, and approximately 50% of patients taking opioids long-term did not report an improvement in chronic LBP [7]. Thus, opioids only have modest effects on pain in patients with chronic LBP and no functional benefits; furthermore, approximately 50% of patients do not tolerate opioids [8].

Nonpharmacological treatments and alternative intervention may be more effective than pharmacological interventions in the management of chronic nonspecific LBP [2]. The guideline from the American College of Physicians and the American Pain Society recommends complementary and alternative interventions, such as exercise therapy, massage, or yoga, for the management of nonspecific LBP [9]. The systematic review of exercises used by LBP patients showed that a variety of exercises appeared to have beneficial effects for LBP and that exercise therapy for chronic LBP appears to be slightly effective in decreasing pain and improving function [10].

The therapeutic application of topical heat is used for the relief of musculoskeletal pain syndromes [11, 12], and thermotherapy using mud can be also recommended to treat patients with chronic LBP [13], neck pain [14–16], or osteoarthritic pain [17]. An appropriate level of pain control by thermotherapy may be required before exercises for pain control in nonspecific LBP. Mud-heat therapy combined with exercise may be more effective for chronic nonspecific LBP relief than exercise only.

To date, no randomized clinical trial (RCT) has been specifically conducted to validate the efficacy of thermointervention using mud with core exercise in patients with nonspecific LBP; thus, the effects of mud-heat in this LBP population remain unclear. The purpose of the present study is to investigate the effects of mud-heat intervention combined with core exercise on pain, functional disability by LBP, and static/dynamic balance in people with chronic LBP. We hypothesized that core exercise following pain control using mud-heat intervention would be more effective than core exercise only in people with chronic LBP.

## 2. Methods

### 2.1. Setting and Participants

This study was conducted as a single-blind, randomized controlled trial and was approved by the Gachon University Institutional Review Board (1044396-201804-HR-105-01). The study was performed in accordance with the protocol, and all subjects provided written informed consent prior to their enrollment in the study.

The inclusion criteria were adults with chronic, nonspecific LBP (pain severity (VAS) > 3/10) either with or without leg pain. Chronic LBP was defined when the duration of the current episode was ≥ 6 months. Participants were permitted to use over-the-counter medication as needed. The exclusion criteria were other concurrent provider-based treatments for LBP, contraindications to study treatment (e.g., clinical spinal instability and inflammatory arthropathies), benign joint hypermobility syndrome, and other serious physical or mental health conditions as determined by self-report, clinical examination, and history.

### 2.2. Experimental Procedures and Intervention

All participants were randomly assigned to either the intervention group or the control group using a stratified randomization method [18]. The participants allocated to the intervention group received mud-heat intervention and then performed the core exercise applied in the previous study [19] for 50 minutes, twice a day, for 4 days, while the control group performed core exercise only.

To apply thermotherapy, moor mud was used in packs [14, 17]; the moor mud was collected at the Chollipo Arboretum, Taean-gun, Chungcheongnam-do, Republic of Korea, in June 2018, and impurities were removed using a 5 mm and 90 *μ*m sieve. Subsequently, 1.5 kg of the moor mud removed from impurities and 300 ml of deep sea water collected from Ulleungdo, East Sea of Korea, were mixed in a zipper bag (30 × 45 cm) and standardized to 1 cm in thickness. The pack was then enveloped by hemp cloth.

The intervention group used a moor mud filled heat pad, whereby participants were instructed to heat the pad to a tolerable temperature and apply it on the lower back twice a day for 30 min over a period of 4 days.

### 2.3. Outcome Measures

This study consisted of two primary (present pain intensity and pain pressure threshold) and three secondary (disability index, muscle properties, and static/dynamic balance) outcome measures.

#### 2.3.1. Pain Intensity

The 100 mm visual analogue scale was used to assess pain intensity at rest and during movement. The visual analogue scale at rest (resting pain) was defined as an unpleasant feeling or pain when patients were still. The visual analogue scale during movement (movement-induced pain) was defined as an unpleasant feeling or pain incurred by full flexion of the trunk [20]. Patients marked their subjective pain intensity at rest and during movement on a 100 mm visual analogue scale table.

#### 2.3.2. Pain Pressure Threshold

The pain pressure threshold (PPT) was measured using a distal algometer (Somedic AB, Farsta, Sweden) with a 1 cm^2^ probe in order to investigate changes in deep tissue nociception of the lower back muscles [21]. The pressure head of the algometer was applied to 5 points of the lumbar area [22]. To assess PPT, the PPT assessment method of a previous study was used [23]. The assessor increased gradually the pressure of the algometer to 10 kPa/s increments until the subjects expressed a pain response, such as a vocalization by pain and a gesture related to pain (hand grasp or eye blink). The mean threshold was calculated for the left- and right-side points.

#### 2.3.3. Low Back Pain Disability

The Oswestry Disability Index (ODI) was used to assess functional disability due to LBP; this consists of 10 items describing the impact of pain on different daily living activities [24]. In the current study, 9 of the 10 items were included, with the exception of sexual function. Each item is scaled on a six-point Likert scale (range 0–5), with 0 indicating no limitation due to pain and 5 indicating that an activity is impossible to perform. The total score ranges from 0 to 45, with a higher score indicating a higher level of disability. The ODI is the most widely used tool for assessing functional outcome in patients with LBP and is recommended for the evaluation of the effectiveness of LBP treatment [25, 26].

#### 2.3.4. Muscle Properties

A handheld myotonometer (Myoton AS, Tallinn, Estonia) was used to measure the mechanical properties of muscle (muscle tone, stiffness, elasticity, relaxation, and creep) of the skeletal muscle at the same region that PPT was measured [27]. The measurements were made after placing the equipment perpendicular to the skin's surface and five repeated measurements were performed. The mean threshold was calculated for the left- and right-side points.

#### 2.3.5. Static/Dynamic Balance

This study used the force plate (Zebris Medical GmbH, Isny, Germany) to measure the center of pressure to analyze the static balance, and MR3 (ver. 8.6) was used to process signals [28]. The patients were placed on a force plate with bare feet. Subjects were kept in an upright posture and their hands were crossed with their arms in a narrow stand position (with ankle and toe touching) in either an eye-open or an eye-closed condition; the measurements were performed three times with a rest interval of 1 minute. The center of pressure (COP) was measured for 40 seconds, and the data for 30 seconds were used, except those for 5 seconds after the start and 5 seconds before the end.

The Timed Up and Go test (TUG) was performed to test dynamic balance; this test requires the performance of sequential motor tasks including standing up, walking straight for 3 m, turning, walking back to the chair, and sitting down [29]. The score for this test was the time required to complete the test, which was measured using a stopwatch. The test was performed twice and the mean was used as a representative value.

### 2.4. Statistical Analysis

Data analyses were performed using SPSS Statistics 21.0 (IBM-SPSS Inc., Chicago, IL). The statistician was blinded to group allocation for all analyses. The Mann–Whitney *U* test and Fisher's exact test were performed in order to analyze the general characteristics between the two groups. The Wilcoxon signed-rank test was used to compare the changes before and after the intervention. The Mann–Whitney *U* test was used to compare the changes between the two groups. A *p* value of <0.05 was considered statistically significant.

## 3. Results

### 3.1. Participants' Characteristics

**Figure 1:**
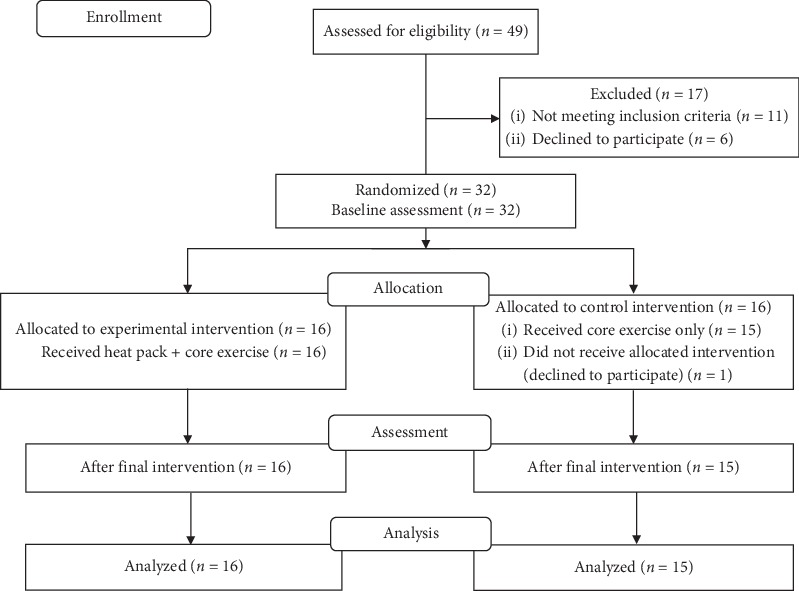
CONSORT flow diagram.

In total, 49 participants were assessed for eligibility and 17 individuals were excluded from participating; 11 did not meet the inclusion criteria and 6 declined to participate. Allocation resulted in baseline comparability between the two groups. In the control group, one declined to participate after allocation. A total of 31 patients completed the study.  Figure 1 shows the selection of participants in this study.

There were no significant differences between the moor therapy and core exercise group (intervention group) and the core exercise group (control group) in terms of the general characteristics of patients with chronic LBP (sex, age, height, weight, duration, and pain intensity) (Table 1). Additionally, there are no significant differences in the prevalues of the outcome variables assessed in this study between the two groups.

### 3.2. Primary Outcome Measure

As shown in Table 2, the intervention group had a significantly decreased pain intensity at rest (*p*=0.001) and during movement (*p*=0.001) before and after intervention. Core exercise significantly decreased the pain intensity at rest (*p*=0.001) and during movement (*p*=0.001). Interestingly, compared to core exercise, moor therapy combined with core exercise significantly improved the pain intensity at rest (*p*=0.048).

With regard to PPT, both groups had significantly increased PPT after intervention (intervention group: L1-M, *p* < 0.001; L3-M, *p*=0.001; L5-M, *p* < 0.001; L1-L, *p* < 0.001; L3-L, *p*=0.001 and control group: L1-M, *p*=0.013; L3-M, *p*=0.023; L5-M, *p*=0.002; L1-L, *p*=0.002; L3-L, *p*=0.003). Compared to core exercise only, moor therapy combined with core exercise led to a significant improvement in PPT (L3-M, *p*=0.009; L5-M, *p*=0.011).

### 3.3. Secondary Outcome Measures

The intervention group showed significant increases in the total ODI score (*p* < 0.001), as well as in pain intensity (*p*=0.001), personal care (*p*=0.001), lifting (*p*=0.001), walking (*p*=0.008), sitting (*p*=0.001), standing (*p*=0.003), sleeping (*p*=0.001), social life (*p*=0.003), and travelling (*p*=0.001). In the control group, the total ODI score was significantly increased (*p*=0.001), and the ODI subcategories were significantly improved, with the exception of pain intensity, *p*=0.002; personal care, *p*=0.005; lifting, *p*=0.007; sitting, *p*=0.007; standing, *p*=0.005; sleeping, *p*=0.021; social life, *p*=0.007; and travelling, *p*=0.002, compared to the core exercise group (Table 3). Moor therapy combined with core exercise showed significant improvements in the total ODI score (*p*=0.001), as well as the pain intensity (*p*=0.005), personal care (*p*=0.011), lifting (*p*=0.002), and walking (*p*=0.037).

As shown in Table 4, the intervention group showed significant differences in muscle tone (L1-M, *p*=0.023; L3-M, *p*=0.046; L5-M, *p*=0.028; and L1-L, *p*=0.005) and stiffness (L1-M, *p*=0.014) after the intervention. The control group also showed significant differences in muscle tone (L1-M, *p*=0.044; L1-L, *p*=0.027) and stiffness (L1-M, *p*=0.017) after the intervention. However, there were no significant differences in any of the muscle properties measured between the two groups.

With regard to static balance, the factors related to COP movement (COP area, length, and velocity) in a standing position with eyes open or closed were assessed. As shown in Table 5, the COP area (*p*=0.002), COP length (*p*=0.004), and COP velocity (*p*=0.012) significantly improved in the eye-open condition, and the values of the COP area (*p*=0.001) significantly decreased in the eye-closed condition after moor therapy with core exercise. The control group showed significant improvements in the area (*p*=0.001), COP length (*p*=0.012), and COP velocity (*p*=0.0015) in the eye-open condition and the area (*p*=0.001) and COP length (*p*=0.027) in the eye-closed condition. With regard to static balance, moor therapy combined with core exercise was significantly more effective than core exercise only (COP length in eye-open condition, *p*=0.026). For dynamic balance assessed by TUG, both groups showed significant improvements in TUG after intervention (intervention, *p*=0.002; control, *p*=0.0011). Compared to core exercise only, moor therapy combined with core exercise significantly improved the dynamic balance assessed by TUG than core exercise only (*p*=0.019).

## 4. Discussion

To the best of our knowledge, this is the first investigation to demonstrate that 8 sessions of mud-heat intervention and core exercise provide benefits that are superior to core exercise only with regard to pain intensity, PPT, disability as a result of LBP, and static/dynamic balance in individuals with chronic LBP. More importantly, the reduction in pain observed with mud therapy combined with core exercise was not only statistically but also clinically meaningful. No significant group effects on muscle properties were documented for mud-heat therapy combined with core exercise vs. core exercise alone. These results may provide evidence to use mud-heat therapy and exercise as an alternative intervention for pain relief and motor improvement in chronic LBP patients.

Medication is the first option in the relief of LBP, but long-term use of medications has side effects such as tolerance or hyperalgesia [7, 8]. Furthermore, the use of medication for pain control can increase the risk of problems such as falls, fractures, and depression [30]. Given these drawbacks, nonpharmacological treatments can be considered as complementary and alternative interventions for the management of chronic nonspecific LBP. An appropriate nonpharmacologic pain control method, and improvement of back muscle strength and stability by core exercise, can be an option for the relief of LBP. The present study showed the effect of mud-heat intervention combined with core exercise for chronic LBP management as a nonpharmacologic and alternative intervention.

Interestingly, this study showed that mud-heat intervention combined with core exercise led to a significant improvement in pain at rest and PPT of low back compared to core exercise alone. In addition, the intervention group was statistically superior to the control group in terms of personal care, lifting, and walking with decreased pain. Previous studies that demonstrated positive effects of mud intervention on chronic musculoskeletal pain, including LBP, support our results [13–17]. The results of our study showed the feasibility of the clinical use of mud-heat intervention for the management of chronic nonspecific LBP. Furthermore, the core exercise alone group also demonstrated a reduction in pain, PPT, and LBP-related disability. Indeed, several studies that investigated the effect of core exercise on pain and sensory and motor function support our results [19, 31, 32]. Core exercise may influence the *β*-endorphin level in patients with chronic nonspecific LBP, and the mechanism of action of the pain-relieving effect of core exercise might be related to an endogenous opioid mechanism [32].

This study also examined the changes in muscle properties of the low back in both groups [33]. Both the intervention and control groups demonstrated significantly decreased muscle tone and stiffness, but elasticity, relaxation, and creep were not significantly different after the intervention. Compared to the core exercise group, the intervention group did not show significant differences in all muscle properties; thus, 8 sessions may not be sufficient to compare the muscle properties between groups. Therefore, long-term and repeated interventions are needed in order to fully determine any changes in muscle tone.

Previous studies have reported that core exercise significantly increased proprioception in LBP [19], and the core stability exercise group showed significant improvements in postural control, assessed by changes in load transfer patterns during [31]. Despite the effect of core exercise on postural control, in this study, mud-heat intervention with core exercise was statistically superior to core exercise in terms of static balance as assessed by COP length. In addition, the intervention group had significantly better dynamic balance than the control group. The significant decrease in pain in the intervention group may be associated with a significant increase of static/dynamic balance in LBP patients [19].

### 4.1. Study Limitations

There are some limitations of the present study. First, since 8 sessions were applied for 4 days, the effect of repeated mud-heat intervention with core exercise over a long-term period is necessary to investigate the clinical use of mud-heat intervention. Second, this pilot study showed significant effects on pain intensity, PPT, LBP-related low back pain, and balance in individuals with chronic LBP; however, the small sample size may limit the generalization of these results.

## 5. Conclusion

The findings of the study demonstrated the efficacy of moor heat combined with core exercise as an alternative therapeutic intervention for pain, disability, and balance in patients with nonspecific LBP. These results recommend the use of moor mud for functional management of LBP patients in the clinic. Since the effect of this intervention on muscle properties was inconclusive, in order to support moor therapy as an evidence-based alternative intervention for nonspecific LBP, further studies with a larger sample size and long-term application of moor are needed.

## Figures and Tables

**Table 1:** General characteristics of participants by study group.

	Intervention group (*n* = 16)	Control group (*n* = 15)	*p*
Sex^†^			
Male	0	3	0.101
Female	16	12	
Age (year)	61.3 ± 9.6	55.8 ± 16.4	0.227
Height (cm)	157.3 ± 4.8	160.9 ± 8.3	0.243
Weight (kg)	59.5 ± 16.7	60.9 ± 13.9	0.069
Duration (year)	10.6 ± 9.7	9.9 ± 5.6	0.921
Pain intensity			
At rest	5.0 ± 1.6	4.9 ± 1.4	0.886
During movement	6.2 ± 1.9	6.1 ± 1.8	0.809

Values are expressed as mean ± SD. ^†^Number of participants.

**Table 2:** Change in pain intensity and pain pressure threshold between groups.

	Intervention (*n* = 16)			Control (*n* = 15)			Group difference		
	Pre	Post	*p*	Pre	Post	*p*	Intervention pre-post	Control pre-post	*p*
Pain intensity									
At rest	4.9 ± 1.4	2.0 ± 1.1.	0.001^*∗*^	4.9 ± 1.5	3.4 ± 1.4	0.001^*∗*^	2.9 ± 1.9	1.6 ± 1.3	0.048^*∗*^
During movement	6.1 ± 1.8	2.3 ± 1.1	0.001^*∗*^	6.0 ± 1.9	3.4 ± 1.5	0.001^*∗*^	3.8 ± 2.1	2.6 ± 1.5	0.099
PPT									
L1-M	4.8 ± 2.4	8.7 ± 3.0	<0.001^*∗*^	4.9 ± 1.3	7.1 ± 3.2	0.013^*∗*^	3.9 ± 2.4	2.2 ± 3.8	0.066
L3-M	4.3 ± 1.8	7.5 ± 2.7	0.001^*∗*^	4.5 ± 1.6	6.5 ± 2.4	0.023^*∗*^	3.2 ± 2.1	2.0 ± 2.2	0.009^*∗*^
L5-M	4.6 ± 2.8	9.0 ± 3.6	<0.001^*∗*^	4.9 ± 2.7	6.2 ± 3.7	0.002^*∗*^	4.4 ± 3.5	1.4 ± 3.2	0.011^*∗*^
L1-L	4.1 ± 2.4	7.9 ± 3.2	<0.001^*∗*^	4.6 ± 2.5	6.3 ± 3.6	0.002^*∗*^	3.8 ± 3.2	1.8 ± 2.7	0.093
L3-L	4.1 ± 2.5	9.2 ± 3.8	0.001^*∗*^	4.4 ± 2.8	6.2 ± 3.7	0.003^*∗*^	5.1 ± 3.7	1.8 ± 2.4	0.060

PPT: pain pressure threshold; L1-M: 2 cm lateral to the L1 spinous process; L3-M: 2 cm lateral to the L3 spinous process; L5-M: 2 cm lateral to the L5 spinous process; L1-L: 5 cm lateral to the L1 spinous process; L3-L: 5 cm lateral to the L1 spinous process. Values are expressed as mean ± SD. ^*∗*^*p* < 0.05.

**Table 3:** Change in low back pain disability.

	Intervention (*n* = 16)			Control (*n* = 15)			Group difference		
	Pre	Post	*p*	Pre	Post	*p*	Intervention pre-post	Control pre-post	*p*
Total score	15.4 ± 5.0	4.5 ± 3.3	<0.001^*∗*^	14.4 ± 4.1	8.9 ± 5.0	0.001^*∗*^	10.9 ± 4.7	5.4 ± 3.0	0.001^*∗*^
Pain intensity	2.6 ± 0.8	0.4 ± 0.5	0.001^*∗*^	2.8 ± 0.8	1.7 ± 1.1	0.002^*∗*^	2.2 ± 0.6	1.1 ± 0.7	0.005^*∗*^
Personal care	1.4 ± 0.8	0.1 ± 0.4	0.001^*∗*^	1.1 ± 0.5	0.6 ± 0.5	0.005^*∗*^	1.3 ± 0.8	0.5 ± 0.5	0.011^*∗*^
Lifting	2.3 ± 1.0	0.8 ± 0.6	0.001^*∗*^	1.6 ± 0.7	1.1 ± 0.8	0.007^*∗*^	1.6 ± 0.8	0.5 ± 0.6	0.002^*∗*^
Walking	0.9 ± 0.7	0.2 ± 0.6	0.008^*∗*^	1.0 ± 0.7	0.8 ± 0.6	0.083	0.7 ± 0.9	0.2 ± 0.4	0.037^*∗*^
Sitting	2.0 ± 0.8	1.0 ± 0.4	0.001^*∗*^	1.6 ± 0.6	1.1 ± 0.7	0.007^*∗*^	1.0 ± 0.7	0.5 ± 0.6	0.085
Standing	1.9 ± 0.9	0.9 ± 0.7	0.003^*∗*^	1.9 ± 0.9	1.2 ± 0.7	0.005^*∗*^	1.1 ± 1.0	0.7 ± 0.7	0.406
Sleeping	1.4 ± 0.9	0.3 ± 0.5	0.001^*∗*^	1.3 ± 0.8	0.7 ± 0.6	0.021^*∗*^	1.2 ± 0.8	0.6 ± 0.8	0.070
Social life	1.3 ± 0.8	0.3 ± 0.6	0.003^*∗*^	1.4 ± 0.6	0.9 ± 0.6	0.007^*∗*^	1.1 ± 1.0	0.6 ± 0.6	0.088
Travelling	1.5 ± 0.6	0.6 ± 0.5	0.001^*∗*^	1.6 ± 0.6	0.8 ± 0.7	0.002^*∗*^	0.9 ± 0.6	0.8 ± 0.6	0.521

Values are expressed as mean ± SD. ^*∗*^*p* < 0.05.

**Table 4:** Change in muscle properties.

	Intervention (*n* = 16)			Control (*n* = 15)			Group difference		
	Pre	Post	*p*	Pre	Post	*p*	Intervention pre-post	Control pre-post	*p*
Tone									
L1-M	19.6 ± 3.4	18.3 ± 2.9	0.023^*∗*^	19.4 ± 3.5	18.9 ± 3.8	0.044^*∗*^	1.3 ± 1.9	0.5 ± 1.9	0.384
L3-M	19.2 ± 4.0	18.0 ± 2.9	0.046^*∗*^	19.0 ± 4.5	18.5 ± 3.9	0.293	1.1 ± 2.2	0.4 ± 2.4	0.649
L5-M	17.7 ± 3.3	16.2 ± 2.4	0.028^*∗*^	17.3 ± 3.2	16.4 ± 2.8	0.096	1.5 ± 2.6	1.0 ± 1.7	0.553
L1-L	17.4 ± 3.0	15.6 ± 2.5	0.005^*∗*^	16.8 ± 2.7	15.0 ± 2.1	0.027^*∗*^	1.8 ± 2.2	1.8 ± 2.4	0.752
L3-L	16.8 ± 2.9	16.2 ± 2.7	0.214	16.3 ± 3.4	16.3 ± 2.9	0.682	0.6 ± 1.7	0.0 ± 1.1	0.312

Stiffness									
L1-M	377.4 ± 64.2	339.9 ± 76.0	0.014^*∗*^	401.3 ± 100.0	387.0 ± 97.0	0.017^*∗*^	22.5 ± 28.7	14.3 ± 17.4	0.243
L3-M	399.5 ± 92.3	379.8 ± 81.3	0.148	397.1 ± 112.2	394.9 ± 114.6	0.955	19.8 ± 40.1	3.2 ± 28.0	0.286
L5-M	345.9 ± 69.8	329.8 ± 68.1	0.115	343.3 ± 87.6	341.1 ± 72.0	0.865	16.2 ± 32.3	2.1 ± 29.3	0.268
L1-L	286.1 ± 46.4	276.0 ± 54.8	0.112	284.3 ± 54.0	271.0 ± 51.7	0.320	10.1 ± 27.4	13.3 ± 29.9	0.722
L3-L	266.6 ± 62.3	250.1 ± 55.6	0.074	269.1 ± 70.1	261.0 ± 65.5	0.649	16.5 ± 30.6	8.1 ± 28.8	0.268

Elasticity									
L1-M	1.9 ± 0.4	1.9 ± 0.3	0.605	1.9 ± 0.5	1.9 ± 0.5	0.820	0.1 ± 0.5	0.0 ± 0.4	0.621
L3-M	1.8 ± 0.4	1.7 ± 0.3	0.088	1.8 ± 0.4	1.7 ± 0.4	0.378	0.1 ± 0.3	0.1 ± 0.2	0.441
L5-M	1.6 ± 0.4	1.4 ± 0.3	0.093	1.7 ± 0.5	1.6 ± 0.5	0.514	0.1 ± 0.3	0.1 ± 0.4	0.514
L1-L	1.4 ± 0.2	1.3 ± 0.2	0.052	1.4 ± 0.4	1.4 ± 0.3	0.670	0.1 ± 0.2	0.1 ± 0.2	0.277
L3-L	1.3 ± 0.2	1.3 ± 0.2	0.088	1.4 ± 0.3	1.3 ± 0.4	0.470	0.1 ± 0.2	0.0 ± 0.2	0.464

Relaxation									
L1-M	14.3 ± 3.9	13.9 ± 3.6	0.469	16.0 ± 4.1	16.2 ± 4.7	0.609	0.4 ± 1.8	−0.2 ± 2.7	0.527
L3-M	15.7 ± 3.9	14.7 ± 3.8	0.093	16.5 ± 4.7	16.4 ± 4.1	0.865	0.9 ± 2.1	0.1 ± 2.1	0.295
L5-M	18.4 ± 4.4	18.0 ± 4.1	0.211	18.1 ± 4.8	17.8 ± 4.0	0.569	0.4 ± 1.1	0.2 ± 1.9	0.905
L1-L	19.6 ± 3.1	19.2 ± 3.3	0.669	20.2 ± 3.5	20.2 ± 4.4	0.320	0.4 ± 2.0	0.0 ± 4.3	0.514
L3-L	21.9 ± 3.6	21.0 ± 3.5	0.079	22.5 ± 4.3	22.3 ± 2.8	0.570	0.9 ± 2.2	0.1 ± 2.2	0.277

Creep									
L1-M	1.0 ± 0.2	0.9 ± 0.2	0.127	1.0 ± 0.2	1.0 ± 0.3	0.932	0.1 ± 0.2	0.0 ± 0.2	0.212
L3-M	0.9 ± 0.3	0.9 ± 0.2	0.737	1.0 ± 0.3	1.0 ± 0.2	0.850	0.0 ± 0.2	0.0 ± 0.1	0.692
L5-M	1.2 ± 0.3	1.1 ± 0.2	0.105	1.2 ± 0.3	1.2 ± 0.2	0.306	0.0 ± 0.1	0.0 ± 0.2	0.621
L1-L	1.2 ± 0.2	1.2 ± 0.2	0.074	1.2 ± 0.2	1.2 ± 0.2	0.172	0.1 ± 0.1	0.0 ± 0.1	0.890
L3-L	1.3 ± 0.2	1.2 ± 0.2	0.125	1.4 ± 0.3	1.3 ± 0.2	0.532	0.1 ± 0.2	0.0 ± 0.2	0.553

L1-M: 2 cm lateral to the L1 spinous process; L3-M: 2 cm lateral to the L3 spinous process; L5-M: 2 cm lateral to the L5 spinous process; L1-L: 5 cm lateral to the L1 spinous process; L3-L: 5 cm lateral to the L1 spinous process. Values are expressed as mean ± SD. ^*∗*^*p* < 0.05.

**Table 5:** Change in static and dynamic balance.

	Intervention (*n* = 16)			Control (*n* = 15)			Group difference		
	Pre	Post	*p*	Pre	Post	*p*	Intervention pre-post	Control pre-post	*p*
Eye open									
COP area (mm^2^)	591.8 ± 360.5	401.9 ± 227.8	0.002^*∗*^	586.6 ± 278.9	515.7 ± 251.5	0.001^*∗*^	189.9 ± 199.5	70.9 ± 57.0	0.097
COP length (mm)	404.9 ± 135.0	337.2 ± 98.7	0.004^*∗*^	401.0 ± 104.6	378.4 ± 106.3	0.012^*∗*^	67.7 ± 69.8	22.5 ± 22.7	0.026^∗^
COP velocity (mm/sec)	13.4 ± 4.5	11.2 ± 2.8	0.012^*∗*^	13.7 ± 4.8	12.7 ± 4.8	0.015^*∗*^	2.2 ± 2.9	1.0 ± 1.2	0.285

Eye close									
COP area (mm^2^)	854.8 ± 680.3	514.4 ± 237.2	0.010^*∗*^	831.1 ± 248.8	593.4 ± 208.7	0.001^*∗*^	340.4 ± 572.4	237.7 ± 172.5	0.502
COP length (mm)	554.7 ± 201.6	457.3 ± 150.8	0.098	538.1 ± 134.8	464.8 ± 129.4	0.027^*∗*^	97.4 ± 159.8	73.3 ± 108.4	0.937
COP velocity (mm/sec)	18.7 ± 6.9	15.5 ± 5.6	0.059	18.1 ± 5.4	16.7 ± 4.7	0.057	3.2 ± 5.9	1.4 ± 2.8	0.501

TUG (sec)	7.9 ± 1.4	6.8 ± 1.1	0.002^*∗*^	7.6 ± 2.1	7.1 ± 2.2	0.011^*∗*^	1.1 ± 0.9	0.5 ± 0.6	0.019^*∗*^

COP: center of pressure; COP area: 95% confidence ellipse area; COP length: center of pressure path length; TUG: Timed Up and Go test. Values are expressed as mean ± SD. ^*∗*^*p* < 0.05.

## Data Availability

The data of this study are available from the corresponding author upon reasonable request.

## Abbreviations

LBP: Low back pain
PPT: Pain pressure threshold
L1-M: 2 cm lateral to the L1 spinous process
L3-M: 2 cm lateral to the L3 spinous process
L5-M: 2 cm lateral to the L5 spinous process
L1-L: 5 cm lateral to the L1 spinous process
L3-L: 5 cm lateral to the L1 spinous process
COP: Center of pressure
TUG: Timed Up and Go test.

## References

[1] Hoy D., Bain C., Williams G. (2012). A systematic review of the global prevalence of low back pain. *Arthritis & Rheumatism*.

[2] Maher C., Underwood M., Buchbinder R. (2017). Non-specific low back pain. *The Lancet*.

[3] O’Sullivan P. B., Mitchell T., Bulich P., Waller R., Holte J. (2006). The relationship beween posture and back muscle endurance in industrial workers with flexion-related low back pain. *Manual Therapy*.

[4] Spenkelink C. D., Hutten M. M., Hermens H. J., Greitemann B. O. (2002). Assessment of activities of daily living with an ambulatory monitoring system: a comparative study in patients with chronic low back pain and nonsymptomatic controls. *Clinical Rehabilitation*.

[5] Luo X., Pietrobon R., Curtis L. H., Hey L. A. (2004). Prescription of nonsteroidal anti-inflammatory drugs and muscle relaxants for back pain in the United States. *Spine*.

[6] Bernstein E., Carey T. S., Garrett J. M. (2004). The use of muscle relaxant medications in acute low back pain. *Spine*.

[7] Deyo R. A., Von Korff M., Duhrkoop D. (2015). Biopsychosocial care for chronic back pain. *BMJ*.

[8] Abdel Shaheed C., Maher C. G., Williams K. A., Day R., McLachlan A. J. (2016). Efficacy, tolerability, and dose-dependent effects of opioid analgesics for low back pain. *JAMA Internal Medicine*.

[9] Chou R., Qaseem A., Snow V. (2007). Diagnosis and treatment of low back pain: a joint clinical practice guideline from the American College of Physicians and the American Pain Society. *Annals of Internal Medicine*.

[10] Hayden J. A., van Tulder M. W., Malmivaara A., Koes B. W. (2005). Exercise therapy for treatment of non-specific low back pain. *Cochrane Database of Systematic Review*.

[11] Nadler S. F., Weingand K., Kruse R. J. (2004). The physiologic basis and clinical applications of cryotherapy and thermotherapy for the pain practitioner. *Pain Physician*.

[12] Geffen S. J. (2003). 3: rehabilitation principles for treating chronic musculoskeletal injuries. *Medical Journal of Australia*.

[13] Weber-Rajek M., Lulińska-Kuklik E., Orłowska K., Czerniachowska I., Radzimińska A., Moska W. (2016). Evaluating the effectiveness of various forms of physical therapy in low back pain treatment. *Trends in Sport Sciences*.

[14] Cramer H., Baumgarten C., Choi K.-E. (2012). Thermotherapy self-treatment for neck pain relief-a randomized controlled trial. *European Journal of Integrative Medicine*.

[15] Wolsko P., Eisenberg D., Davis R., Kessler R., Phillips R. (2003). Patterns and perceptions of care for treatment of back and neck pain: results of a national survey. *Spine*.

[16] Borghouts J., Janssen H., Koes B., Muris J., Metsemakers J., Bouter L. (1999). The management of chronic neck pain in general practice: a retrospective study. *Scandinavian Journal of Primary Health Care*.

[17] Tefner I. K., Gaál R., Koroknai A. (2013). The effect of Neydharting mud-pack therapy on knee osteoarthritis: a randomized, controlled, double-blind follow-up pilot study. *Rheumatology International*.

[18] Zelen M. (1974). The randomization and stratification of patients to clinical trials. *Journal of Chronic Diseases*.

[19] Kim T. H., Kim E.-H., Cho H.-Y. (2015). The effects of the CORE programme on pain at rest, movement-induced and secondary pain, active range of motion, and proprioception in female office workers with chronic low back pain: a randomized controlled trial. *Clinical Rehabilitation*.

[20] Weiner D. K., Rudy T. E., Kim Y. S., Golla S. (2004). Do medical factors predict disability in older adults with persistent low back pain?. *Pain*.

[21] Potter L., McCarthy C., Oldham J. (2006). Algometer reliability in measuring pain pressure threshold over normal spinal muscles to allow quantification of anti-nociceptive treatment effects. *International Journal of Osteopathic Medicine*.

[22] Hirayama J., Yamagata M., Ogata S., Shimizu K., Ikeda Y., Takahashi K. (2006). Relationship between low-back pain, muscle spasm and pressure pain thresholds in patients with lumbar disc herniation. *European Spine Journal*.

[23] Hahm S.-C., Suh H. R., Cho H.-y. (2019). The effect of transcutaneous electrical nerve stimulation on pain, muscle strength, balance, and gait in individuals with dementia: a double blind, pilot randomized controlled trial. *European Journal of Integrative Medicine*.

[24] Fairbank J., Couper J., Davies J., O’Brien J. (1980). The Oswestry low back pain disability questionnaire. *Physiotherapy*.

[25] Bombardier C. (2000). Outcome assessments in the evaluation of treatment of spinal disorders. *Spine*.

[26] Deyo R. A., Andersson G., Bombardier C. (1994). Outcome measures for studying patients with low back pain. *Spine*.

[27] Leonard C. T., Deshner W. P., Romo J. W., Suoja E. S., Fehrer S. C., Mikhailenok E. L. (2003). Myotonometer intra-and interrater reliabilities11a commercial party with a direct financial interest in the results of the research supporting this article has conferred or will confer a financial benefit upon one or more of the authors. *Archives of Physical Medicine and Rehabilitation*.

[28] Bauer C., Gröger I., Rupprecht R., Gassmann K. G. (2008). Intrasession reliability of force platform parameters in community-dwelling older adults. *Archives of Physical Medicine and Rehabilitation*.

[29] Podsiadlo D., Richardson S. (1991). The timed “up & go”: a test of basic functional mobility for frail elderly persons. *Journal of the American Geriatrics Society*.

[30] Solomon D. H., Rassen J. A., Glynn R. J. (2010). The comparative safety of opioids for nonmalignant pain in older adults. *Archives of Internal Medicine*.

[31] Muthukrishnan R., Shenoy S. D., Jaspal S. S., Nellikunja S., Fernandes S. (2010). The differential effects of core stabilization exercise regime and conventional physiotherapy regime on postural control parameters during perturbation in patients with movement and control impairment chronic low back pain. *BMC Sports Science, Medicine and Rehabilitation*.

[32] Paungmali A., Joseph L. H., Punturee K., Sitilertpisan P., Pirunsan U., Uthaikhup S. (2018). Immediate effects of core stabilization exercise on *β*-endorphin and cortisol levels among patients with chronic nonspecific low back pain: a randomized crossover design. *Journal of Manipulative and Physiological Therapeutics*.

[33] Hu X., Lei D., Li L. (2018). Quantifying paraspinal muscle tone and stiffness in young adults with chronic low back pain: a reliability study. *Scientific Reports*.

